# *Panax ginseng* C.A. meyer alleviates benign prostatic hyperplasia while preventing finasteride-induced side effects

**DOI:** 10.3389/fphar.2023.1039622

**Published:** 2023-01-12

**Authors:** Ja Yeon Park, Woo Yong Park, Gahee Song, Se Jin Jung, Beomsu Kim, Minji Choi, Sang Hee Kim, Jinbong Park, Hyun Jeong Kwak, Kwang Seok Ahn, Jun Hee Lee, Jae-Young Um

**Affiliations:** ^1^ Department of Science in Korean Medicine, Graduate School, Kyung Hee University, Seoul, Republic of Korea; ^2^ Department of Pharmacology, College of Korean Medicine, Kyung Hee University, Seoul, Republic of Korea; ^3^ Department of Life Science, College of Natural Sciences, Kyonggi University, Seoul, Republic of Korea; ^4^ Department of Science in Korean Medicine, Kyung Hee University, Seoul, Republic of Korea; ^5^ Department of Sasang Constitutional Medicine, College of Korean Medicine, Kyung Hee University, Seoul, Republic of Korea

**Keywords:** dihydrotestosterone, androgen receptor, 5α-reductase 2, apoptosis, sperm loss

## Abstract

*Panax ginseng* C.A. Meyer, a widely used traditional medicine in East Asia, shows many beneficial effects on immune function, male erectile dysfunction, cancer, excessive oxidants, and aging issues. However, its effect on benign prostatic hyperplasia (BPH) and its potential in the treatment of side effects related to finasteride (Fi), an FDA-approved drug for BPH, are less known. This study aimed to verify the therapeutic effects of a water extract of *P. ginseng* (PGWE) on BPH in testosterone propionate (TP)-induced BPH rats and TP-treated RWPE-1 human epithelial cells, and the inhibitory potential on the Fi-induced side effects is also explored. In the TP-induced BPH rat model, PGWE alleviated the pathological markers of BPH such as weight and epithelial thickness of the prostate, and the serum level of dihydrotestosterone. PGWE downregulated androgen-related BPH factors such as 5α-reductase 2 and androgen receptor. PGWE also showed prostatic cell apoptosis accompanied by increased expression of Bax and decreased expression of Bcl-xL and cleaved-caspase 3, respectively, in addition to increasing mitochondrial dynamics in both *in vivo* and *in vitro* BPH models. Notably, reduced sperm count, one of the serious side effects of Fi, in the epididymis of BPH rats was recovered with PGWE treatment, suggesting less toxicity to sperm development by PGWE. PGWE also protected against Fi-induced sperm loss when PGWE was administered in combination with Fi without compromising the therapeutic effects of Fi on BPH. Based on these findings, we propose that PGWE could be an alternative therapeutic agent for BPH.

## Introduction

Benign prostatic hyperplasia (BPH) is the most common chronic urinary tract disease in elderly men (Thorpe and Neal, 2003). According to epidemiological investigations, the incidence of BPH gradually increases with age, with incidence of 50% at the age of 50 and incidence of 90% at the age of 80 or older (Egan, 2016). BPH is characterized by an abnormal increase in the proliferation of smooth muscle cells, stromal cells, and epithelial cells in the prostate (Marszalek et al., 2009). When the prostate is enlarged, it compresses the urethra, resulting in low urinary tract symptoms (LUTS), such as weak urine stream, frequent urination, residual urine, urgency, and leakage of urine (Mobley et al., 2015). These symptoms can degrade the patient’s quality of life. Previous studies have reported that various factors, including sex hormone changes, aging, and growth factors, are associated with the development and progression of BPH, but the exact molecular mechanism of BPH is not known yet, and further studies are needed (Roehrborn, 2008).

Although the exact mechanism of BPH is not clear, among causes, one of most well-known is changes in androgen levels (Ho and Habib, 2011). Testosterone is converted by 5α-reductases to produce dihydrotestosterone (DHT) which contributes to the development of BPH. DHT has a significantly higher binding affinity with the androgen receptor (AR), and thus it binds to AR before testosterone. This binding promotes translocation to the nucleus by causing the decomposition of heat shock proteins and the phosphorylation of AR (La Vignera et al., 2016). As a result, the proliferation of epithelial cells or stromal cells in the prostate is accelerated, resulting in an imbalance in prostate proliferation and apoptosis.

The most effective way to treat BPH is transurethral resection (Nickel et al., 2010). It is a surgical procedure that involves cutting away a section of the prostate. But this surgical method can cause complications such as bleeding or urinary incontinence, and there is a high risk to the elderly (McAllister et al., 2003). Therefore, pharmacological treatments have been widely used in recent years. There are two kinds of medicines used to treat BPH: a 5α-reductase inhibitor and an alpha blocker. 5α-reductase inhibitors such as finasteride (Fi) and dutasteride suppress proliferation of the prostate by reducing the conversion of testosterone to DHT (Paolone, 2010). Alpha-blockers allow urine to flow more easily by relaxing the smooth muscles in the prostate and reducing the muscle tension in the bladder (Schwinn and Roehrborn, 2008). Although these drugs have proven useful in alleviating symptoms of BPH, they are accompanied by several side effects. For example, 5α-reductase inhibitors cause serious allergic reactions such as dyspnea, rash, depression, and erectile dysfunction (McConnell et al., 1998). Because these synthetic materials have various risks, many studies are being conducted to develop new drugs from natural sources. Currently, Serenoa repens, known as saw palmetto, is a natural compound produced from a fruit extract of a type of palmtree that has recently been widely used to treat BPH through anti-androgenic, proapoptotic, and anti-inflammatory effects. However, it is also reported to have minor side effects such as abdominal pain, diarrhea, nausea, fatigue, headache, decreased libido, and rhinitis (Agbabiaka et al., 2009).

*Panax ginseng* C.A. Meyer is one of the most widely used herbal medicines in East Asia (Kiefer and Pantuso, 2003). It is a medicinal plant belonging to the Araliaceae family, of which the roots are mainly used. The roots of *P. ginseng* contain numerous components including ginsenoside, flavonoid, and polysaccharides (Ru et al., 2015). *Panax* is a term with Greek etymology that combines Pan, which means “everything”, and Axos, which means “medicine”, together meaning “panacea” (Jia and Zhao, 2009). As the scientific name suggests, *P. ginseng* is excellent in preventing diverse diseases. According to previous studies, it is also reported that *P. ginseng* is effective in improving brain function, immune function, and male erectile dysfunction, and has anti-cancer, antioxidant, and anti-aging roles (Yun and Choi, 1998; Chung et al., 2021; Hyun et al., 2021). Although *P. ginseng* exhibits several beneficial effects in various diseases, its role in the regulation of BPH has not been fully explored.

The aim of this study was to investigate the effects of *Panax ginseng* C.A. Meyer (PGWE) on BPH by using testosterone propionate (TP)-induced BPH rats and TP-treated RWPE-1 cells and to determine whether PGWE has potential as an alternative agent for BPH treatments*.*

## Materials and methods

### Chemical reagents

Roswell Park Memorial Institute (RPMI) medium, penicillin-streptomycin, and fetal bovine serum (FBS) were purchased from Gibco BRL (Grand Island, NY, United States). TP was purchased from Wako Pure Chemical Industries (Osaka, Japan), and Fi was purchased from Tokyo Chemical Industries (Tokyo, Japan). An electrochemiluminescence (ECL) kit was obtained from GE Healthcare Life Sciences (Seoul, Korea).

### Antibodies

BAX (2772S), BCL-XL (2764S), caspase-3 (14220S), PARP (9532S), and β-actin (3700S) were obtained from Cell Signaling Technology (Beverly, MA, United States). MFN1 (sc-166644), DRP1 (sc-271583), AR (sc-816, sc-7305), and PSA (sc-7316) were purchased from Santa Cruz Biotechnology (Dallas, Tx, United States). SRD5A2 (orb101414) was obtained from Biorbyt (Cambridge, United Kingdom).

### Preparation of the PGWE

*Panax ginseng* C.A. Meyer was purchased from Omniherb (Daegu, Korea). PGWE was obtained by extracting *P. ginseng* C.A. Meyer in hot water at 100°C for 3 h, followed by filtering (No 4, Whatman, Kent, United Kingdom). After being freeze-dried in a vacuum, it was diluted to a concentration of 2.5 mg/mL using distilled water. The solution was filtered through a 0.22 μm syringe filter, and then stored at −20°C until usage. The dose of administration was determined based on the clinical use of *P.ginseng*. Typically, *P.ginseng* is prescribed within the range of 0.5g–2 g per day (World Health Organization, 1999), and thus we used 80 mg/kg in rats as the maximal dose according to human use.

### Animal experiment

Six-week-old male Sprague Dawley (SD) rats (body weight 180–200 g) were purchased from the DaeHan Experimental Animal Center (Dae-Han Biolink, Eumsung, South Korea). The rats were housed in a pathogen-free room maintained at 23°C ± 2°C and relative humidity of 70% with an alternating 12 h light/dark cycle. Water and standard laboratory diet (CJ Feed Co., Ltd., Seoul, South Korea) were provided *ad libitum*. BPH was induced as previously described (Park et al., 2019). To induce BPH, TP was administered for 8 weeks during the entire experimental period. Briefly, after daily subcutaneous injections of TP (5 mg/kg/d) for 4 weeks in the inguinal region of rats, the rats were divided into four groups with six animals in each group: 1) a normal control group (NC): ethanol with corn oil, 2) a BPH group: TP with corn oil, 3) a positive control group: Fi (1 mg/kg) with TP (5 mg/kg), and 4) experimental group: PGWE (80 mg/kg) with TP (5 mg/kg). To evaluate the side effect of Fi, secondary experimental group was divided (n = 4/each group) as follows: 1) normal control group (NC), 2) BPH group, 3) Fi-treated (Fi) group, and 4) PGWE in the presence of Fi (PGWE + Fi) group. PGWE (*p.o.*) and Fi (inguinal) were administered once daily for 4 weeks after the treatment of TP for 4 weeks. After the final treatment, the animals were fasted overnight and euthanized using CO_2_. Blood samples were obtained from the caudal vena cava. The blood containing tubes remained at RT for 2 h and then sera were separated by centrifuging at 3,000 × *g* for 20 min at 4°C. The serum was stored at −80°C until used in assays. The intact prostate tissue was carefully dissociated and removed, washed with PBS, and then weighed. Relative prostate weight was calculated as the ratio of prostate weight (mg) to body weight (100 g).

### Hematoxylin and eosin staining

The preparation of prostate tissue sections and H&E staining were performed as described previously (Park et al., 2021). Briefly, prostate tissue was washed in PBS and fixed in 10% formalin for 2 weeks. The tissues were then embedded in paraffin. The tissue sections were deparaffinized in xylene, rehydrated with ethanol/water, and then stained with H&E. Microscopic examinations were performed, and photomicrographs were taken using an EVOSR Cell Imaging system (Thermo Scientific, Carlsbad, CA, United States). Epithelial thickness was measured using the ImageJ software program (National Institute of Health, Bethesda, MD, United States).

### Western blot analysis

Homogenized prostate tissues and harvested RWPE-1 cells were lysed through radioimmunoprecipitation assay (RIPA) buffer (Cell Signaling Technology, Danvers, MA, United States) on ice for 30 min, and then, insoluble materials were removed by centrifugation at 13,000 rpm for 20 min at 4°C. The lysates were resolved by sodium dodecyl sulfate (SDS)-polyacrylamide gel electrophoresis and transferred onto a polyvinylidene difluoride (PVDF) membrane. The membranes were then blocked in 5% skim milk and incubated with the respective primary antibody (1:1000) overnight at 4°C followed by incubation with horseradish peroxidase (HRP)-conjugated secondary antibody (1:5000) for 1 h at room temperature. The protein signals were detected using an ECL advance kit. The chemiluminescent intensities of protein signals were quantified using ImageJ software (National Institute of Health).

### JC-1 staining

For immunofluorescent staining of JC-1, the cells were stained with 10 µM JC-1 reagent for 30 min at 37°C. For flow cytometry analysis of JC-1, the ratio of red/green fluorescence (polymer) was measured after staining the cells with JC-1 dye (MedChemExpress, LLC., Monmouth Junction, NJ, United States) according to the manufacturer’s instructions.

### DCFDA staining

The intracellular ROS levels were quantified with oxidation sensitive DCFDA reagent. A portion (10 μL) of 10 μM DCFDA was added and the cells were kept at 37°C for 10–30 min in the dark. Cells were then visualized under an EVOSR Cell Imaging system (Thermo Scientific, Carlsbad, CA, United States).

### Immunofluorescence staining

The cells and tissues were fixed using 10% formalin and blocked with 5% BSA for 1 h. Afterward, the cells and tissue were incubated with the indicated primary antibodies (anti-AR, 1:50 in 5% BSA) overnight at 4°C. After washing, the cells and tissue were incubated with Alexa Flour 488- or 633-conjugated secondary antibody (1:1000), and the fluorescence was detected using an EVOSR Cell Imaging system (Thermo Scientific, Carlsbad, CA, United States).

### Cell culture

The normal human prostatic epithelial cell line RWPE-1 was obtained from the American Type Culture Collection (Manassas, VA, United States). RWPE-1 cells were cultured in Roswell Park Memorial Institute medium (RPMI) (Gibco, Big Cabin, OK, United States) supplemented with 100 mg/mL penicillin/streptomycin (HyClone, Logan, UT, United States) and 10% FBS (Sigma-Aldrich Inc). After 24 h of incubation, the culture media was replaced with fresh media containing 4 µM of TP to induce cell proliferation. PGWE and Fi were then supplemented after 30 min.

### Cell cytotoxicity assay

The RWPE-1 cells were seeded (3 × 10^4^ cells per well) on 96-well plates and incubated in RPMI plus 10% FBS for 24 h. The cells were then incubated in fresh media containing various concentrations of PGWE (0–5 mg/mL) for an additional 24 h. Cell viability was measured using a Quanti-MAX^™^ WST-8 cell viability assay kit from Biomax Corporation (Seoul, Korea). Before measuring the viability, the media were removed, replaced with 200 µL of fresh RPMI plus 10% FBS medium, and 10 µL of Quanti-MAX^™^ (WST-8) was added to each well. The cells were then incubated for 1 h. The absorbance was measured at 490 nm in a VERSAmax microplate reader (Molecular Devices, Sunnyvale, CA, United States) to determine the formazan concentration, which is proportional to the number of live cells.

### RNA isolation and real-time reverse transcription-polymerase chain reaction (RT-PCR)

RNA isolation and real-time RT-PCR were performed as previously described (Park et al., 2022). Briefly, the total RNA was obtained using GeneAllR RiboEX Total RNA extraction (GeneAll Biotechnology, Seoul, Korea). The relative gene expressions were calculated based on the comparative CT method using StepOne software v2.1 (Applied Biosystems, Foster City, CA, United States). The mRNA expression of Gapdh was used as an endogenous control. The primers used in this study were: 5AR2 (F: 5′-TCC​CGC​TTG​GCC​TTT​TG-3′, R: 5′-GCC​GTT​ACC​CTC​CTT​GTT​TTC-3′), AR (F: 5’ -TCA​CCC​CCC​AGG​AAT​TCC -3′, R: 5′-ATG​ATA​CGA​TCG​AGT​TCC​TTG​ATG -3′), and GAPDH (F: 5′-AAC​TTT​GGC​ATT​GTG​GAA​GG-3′, R: 5′-GGA​TGC​AGG​GAT​GAT​GTT​CT-3′).

### Epididymal sperm count analysis

The cauda epididymis was removed, slashed twice, and placed into 4 mL of PBS for 1 h to let the sperm diffuse. Sperm-containing PBS was then mixed with the same amount of methanol, and an epididymal sperm count was carried out using a hemocytometer under an EVOSR cell imaging system (Thermo Scientific, Carlsbad, CA, United States). Thirty samples from each group were used in the evaluation.

### Liquid chromatography/mass spectrometry (LC/MS) analysis of PGWE

We used LC/MS analysis to identify the chemical profiling of PGWE. Chromatographic separation of the aliquots was performed by an Agilent 1290 infinity LC (Agilent Technologies, Palo Alto, United States) using an Agilent Eclipse Plus C18 column (2.1 × 50 mm, 1.8 µm) and a mobile phase composed of 0.1% formic acid in water (A) and 0.1% formic acid in acetonitrile (B). The gradient program was as follows: 0–3 min, 5% B; 3–13 min, 5%–80% B; 13–15 min, 80% B; 15–17 min, 80%–5% B; and 17–20 min, 5% B. The flow rate was 0.3 mL/min, and the injection volume was 1 μL, which was injected into the column using a thermostatted HiP-ALS autosampler. Separated peaks were analyzed using an Agilent 6550 Q-TOF (Agilent Technologies), which provided high-resolution mass measurement. The instrument was equipped with a Jet Stream ESI source. The ESI spray voltage was set to 4000 V for positive ion mode and 3500 V for negative ion mode. Mass spectra were acquired in the 100–1000 m/z range.

### Statistical analysis

All data are expressed as mean ± SEM from independent experiments. Statistical differences were evaluated using the Student’s t-test or one-way ANOVA, and a subsequent post-hoc test *via* Prism 8 (GraphPad Software, San Diego, CA, United States). Values of p* < 0.05 and p** < 0.01 were considered statistically significant.

## Results

### Chemical profiling of PGWE using LC/MS analysis

**FIGURE 1:**
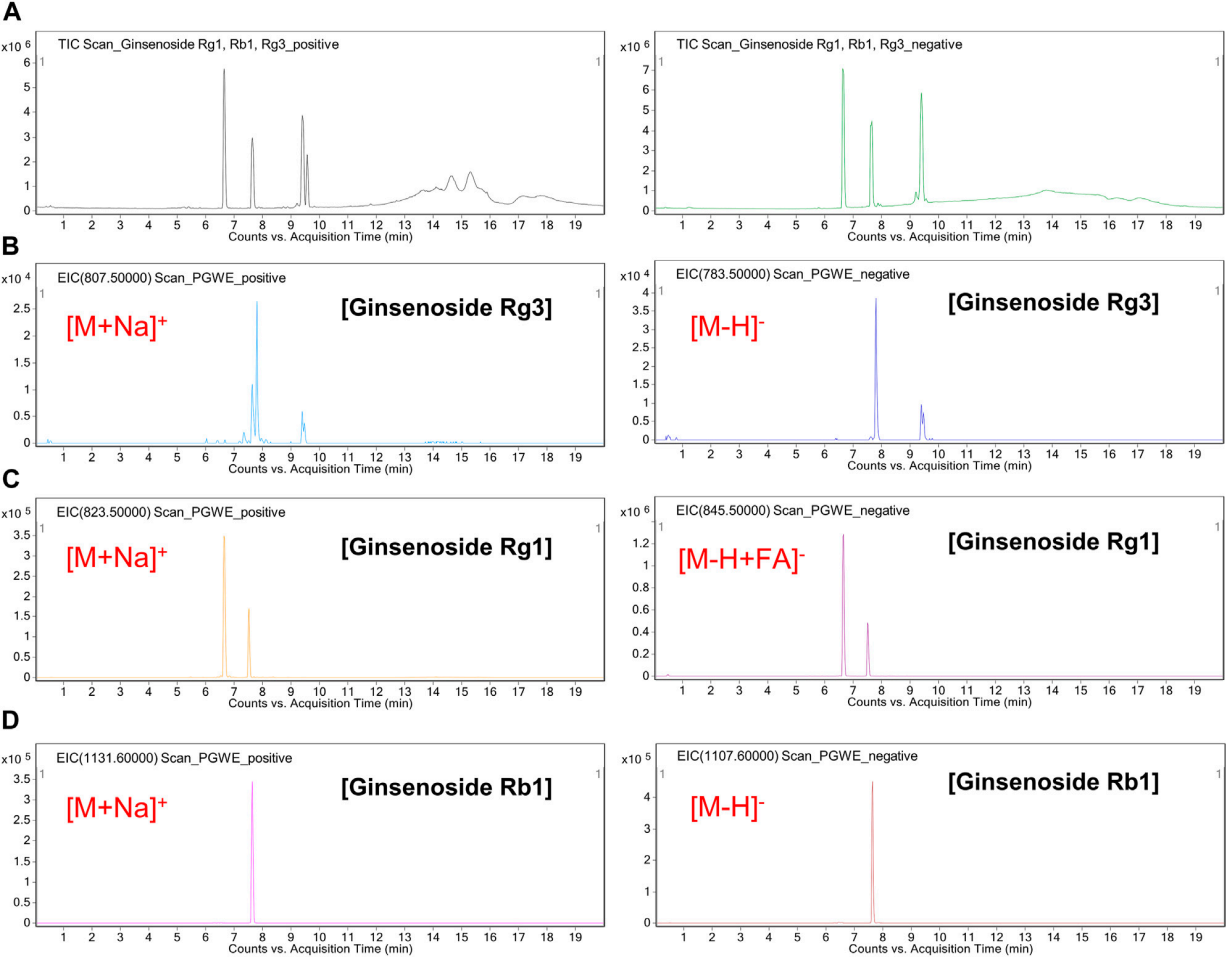
LC/MS analysis of PGWE **(A)** Positive or negative TIC of a cocktail of ginsenoside (Rg1, Rb1, Rg3) was analyzed using the ESI method **(B–D)** Positive or negative EIC of the PGWE was obtained at m/z 783.5 or at m/z 807.5, m/z 845.5 or m/z 823.5, m/z 1107.6 or m/z 1131.6, respectively. PGWE, *Panax ginseng* C. A. Meyer water extract; TIC, total ion chromatogram; ESI, electrospray ionization; EIC, extracted ion chromatogram.

To provide a chemical profile of PGWE, we conducted a LC/MS analysis (Figure 1). Based on the results, we confirmed that PGWE contains ginsenoside Rg3, Rg1, and Rb1, which already have been identified as the main ingredients of *P. ginseng* C.A. Meyer.

### Effects of PGWE on the enlargement of the prostate and histological changes in BPH

**FIGURE 2:**
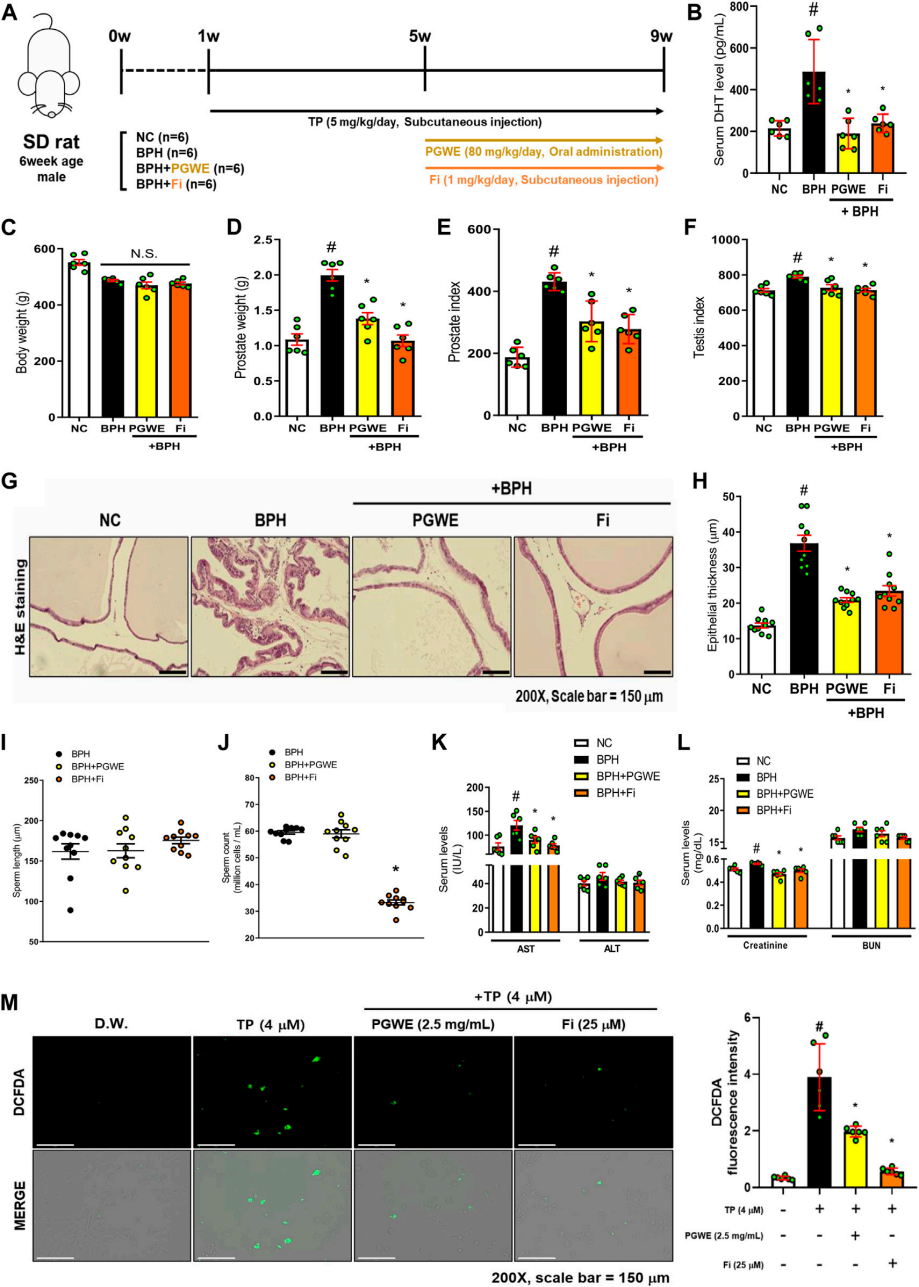
PGWE alleviates pathological signs of BPH in TP-induced BPH rats **(A)** Experimental scheme of *in vivo* study **(B)** Serum DHT levels were analyzed using ELISA kits **(C)** Body and **(D)** prostate weights were assessed **(E)** Prostate index was calculated by prostate weight (mg)/body weight (100 g) **(F)** Testis index was calculated by testis weight (mg)/body weight (100 g) **(G)** H and E staining analysis was performed using prostate tissues of TP-induced BPH rats (magnification × 200, scale bar 150 µm) **(H)** Based on H and E staining, the epithelial thickness was measured using ImageJ software **(J)** Number of sperm extracted from the epididymis of rats and **(I)** length of sperm were calculated using ImageJ software **(K, L)** Serum levels of AST, ALT, creatinine, and BUN were measured **(M)** ROS levels were detected by fluorescence microscopy after DCFDA staining in TP-treated RWPE-1 cells. All data are expressed as mean ± SEM of independent experiments. Statistical differences were calculated by one-way ANOVA with post-hoc Tukey’s test. ^#^ *p* < 0.05 vs NC groups or D.W.-treated RWPE-1 cells; ^∗^ *p* < 0.05 vs BPH group or TP-treated RWPE-1 cells. NC, normal control; BPH, benign prostatic hyperplasia; TP, testosterone propionate; PGWE, *Panax ginseng* C. A. Meyer water extract; Fi, finasteride; D.W., distilled water.

To evaluate the therapeutic effects of PGWE, we established an experimental BPH rat model by the administration of TP. Twenty-four SD rats were used in the animal experiment and were divided into four groups: normal control (NC), TP-treated (BPH), PGWE-treated (PGWE), and Fi-treated (Fi) group. As mentioned, BPH was induced by injecting TP for 8 weeks into the three groups (that is, except for the NC group). From Week 5, the PGWE and Fi groups were administered either PGWE or Fi, respectively, for 4 weeks. The detailed experimental scheme is shown in Figure 2A. As shown in Figure 2C, there was no significant difference in body weight among the groups. The prostate weight and prostate index were significantly increased in the BPH group, whereas a decrease was observed in the PGWE and Fi groups (Figures 2D, E). Furthermore, the testis index was decreased by PGWE and Fi treatment compared to the BPH group (Figure 2F).

Prostate tissue fragments of the BPH rats contain a great deal of information, such as the enlargement of epithelial thickness and infiltration of the luminal area that occurred due to increased DHT. Therefore, we investigated the histological changes to determine if PGWE relieved the pathological signs. As shown in Figures 2G, H, the pathological changes were alleviated by PGWE and Fi treatment. Fi, which is already used as a treatment for BPH, has side effects such as decreased sexual functions (Yu et al., 2020). Our experiment also showed that the Fi group had a decrease in sperm count compared to the BPH group (Figure 2J). However, in the PGWE group, the sperm count was similar to that of the BPH group. In contrast, there was no difference among the groups in sperm length (Figure 2I).

DHT is a crucial factor in the proliferation of prostate epithelial cells (Gao et al., 2005). Therefore, we analyzed the serum level of DHT to determine how it changes with PGWE treatment (Figure 2B). As expected, the DHT levels of the BPH groups were significantly higher than those of the NC groups and was decreased by PGWE treatment. Additionally, to determine whether PGWE damaged the liver of the rats, we analyzed the serum levels of AST, ALT, creatinine, and BUN. As shown in Figures 2K, L, PGWE and Fi decrease the factors that increased in TP-induced BPH rats.

ROS is a product that is naturally generated in the body during the metabolic process of oxygenation. However, excessive production of ROS can be harmful to the body (Hamid et al., 2011). According to previous reports, it is known that ROS levels are increased in BPH (Li et al., 2019). Therefore, DCFDA staining was performed in TP-treated RWPE-1 cells to investigate the changes in ROS levels induced by PGWE treatments. As shown in Figure 2M, ROS was significantly decreased by PGWE treatment.

From these results, we confirmed that PGWE alleviates typical pathological symptoms of BPH without any side effects as seen in finasteride.

### The inhibitory effect of PGWE on androgen signaling in BPH

**FIGURE 3:**
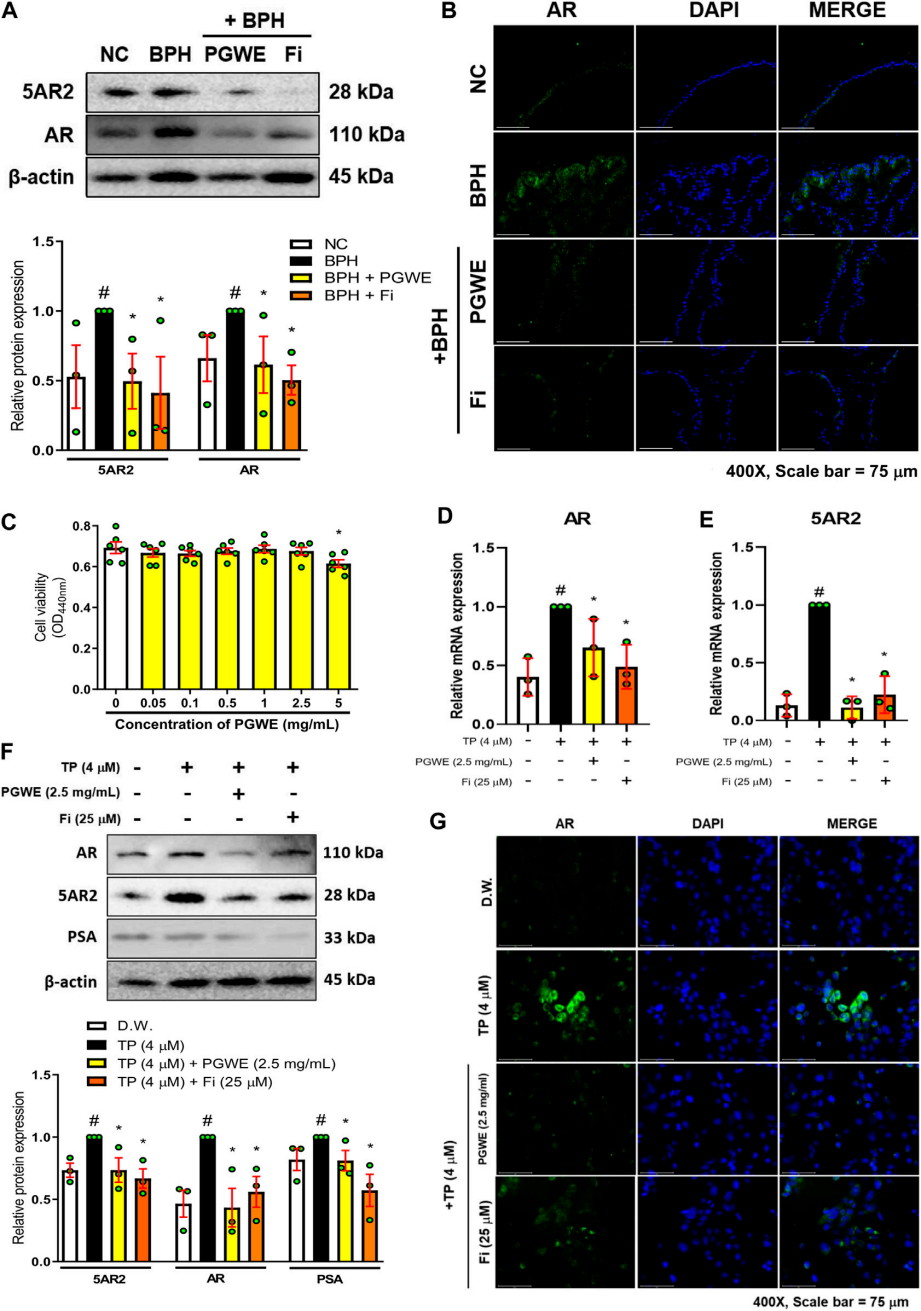
PGWE reduces 5AR2 and AR levels in prostate tissues of BPH rats and TP-treated RWPE-1 cells **(A)** The expressions of AR and 5AR2 in prostate tissues of TP-induced BPH rats were analyzed by western blotting. The loading control was β-actin **(B)** An immunofluorescence assay of AR was conducted in the prostate tissue (magnification × 400, scale bar 75 µm) **(C)** RWPE-1 cells were treated with various concentrations of PGWE for 24 h to assay cell viability **(D, E)** Relative mRNA expressions of 5AR2 and AR were determined by RT-PCR **(F)** The protein expressions of AR, 5AR2, and PSA in RWPE-1 cells were analyzed by western blotting and β-actin was used as the loading control **(G)** Immunofluorescence assay of AR was conducted in RWPE-1 cells (magnification × 400, scale bar 75 µm). All data are expressed as mean ± SEM of independent experiments. Statistical differences were evaluated using an unpaired *t*-test and a subsequent *post hoc* one-tailed *Mann-Whitney U* test. ^#^ *p* < 0.05 vs. NC groups or DW-treated RWPE-1 cells; ^∗^ *p* < 0.05 vs. BPH group or TP-treated RWPE-1 cells. NC, normal control; BPH, benign prostatic hyperplasia; TP, testosterone propionate; PGWE, *Panax ginseng* C. A. Meyer water extract; Fi, finasteride; D.W., distilled water.

The excessive presence of testosterone in the blood was converted into a large amount of DHT by 5α-reductases in the prostate and the synthesized DHT binds to the AR in prostate cells, causing enlargement of the prostate. As expected, the protein level of 5AR2 and AR in prostate tissues of the BPH group was increased by treating TP (Figure 3A). However, the increased protein levels of 5AR2 and AR were inhibited by PGWE or Fi treatments. Through immunofluorescence staining using prostate tissue from TP-induced BPH rats, we checked once more that PGWE has the effect of inhibiting AR (Figure 3B).

We also confirmed the effectiveness of PGWE on BPH through an *in vitro* BPH model. First, to find appropriate concentrations of PGWE for the experiments, we measured cell viability in RWPE-1 cells (Figure 3C). As a result, we selected 2.5 mg/mL as the treatment concentration of PGWE, which does not show cytotoxicity in RWPE-1 cells. In line with the results of animal experiments, mRNA expression of 5AR2 and AR in TP-treated RWPE-1 cells increased, and then were decreased by PGWE or Fi treatments (Figures 3D, E). Consistently, the protein expressions of 5AR2, AR, and PSA were also decreased by PGWE or Fi treatments (Figure 3F). Furthermore, we confirmed that PGWE treatment reduced the increased cytosolic/nuclear AR expression in TP-treated RWPE-1 cells (Figure 3G). These findings suggest that PGWE interferes with androgen signaling in both rats and human prostate epithelial cells, thereby inhibiting androgen-mediated cell proliferation of the prostate.

### Effect of PGWE on mitochondrial dynamics and apoptosis in BPH

**FIGURE 4:**
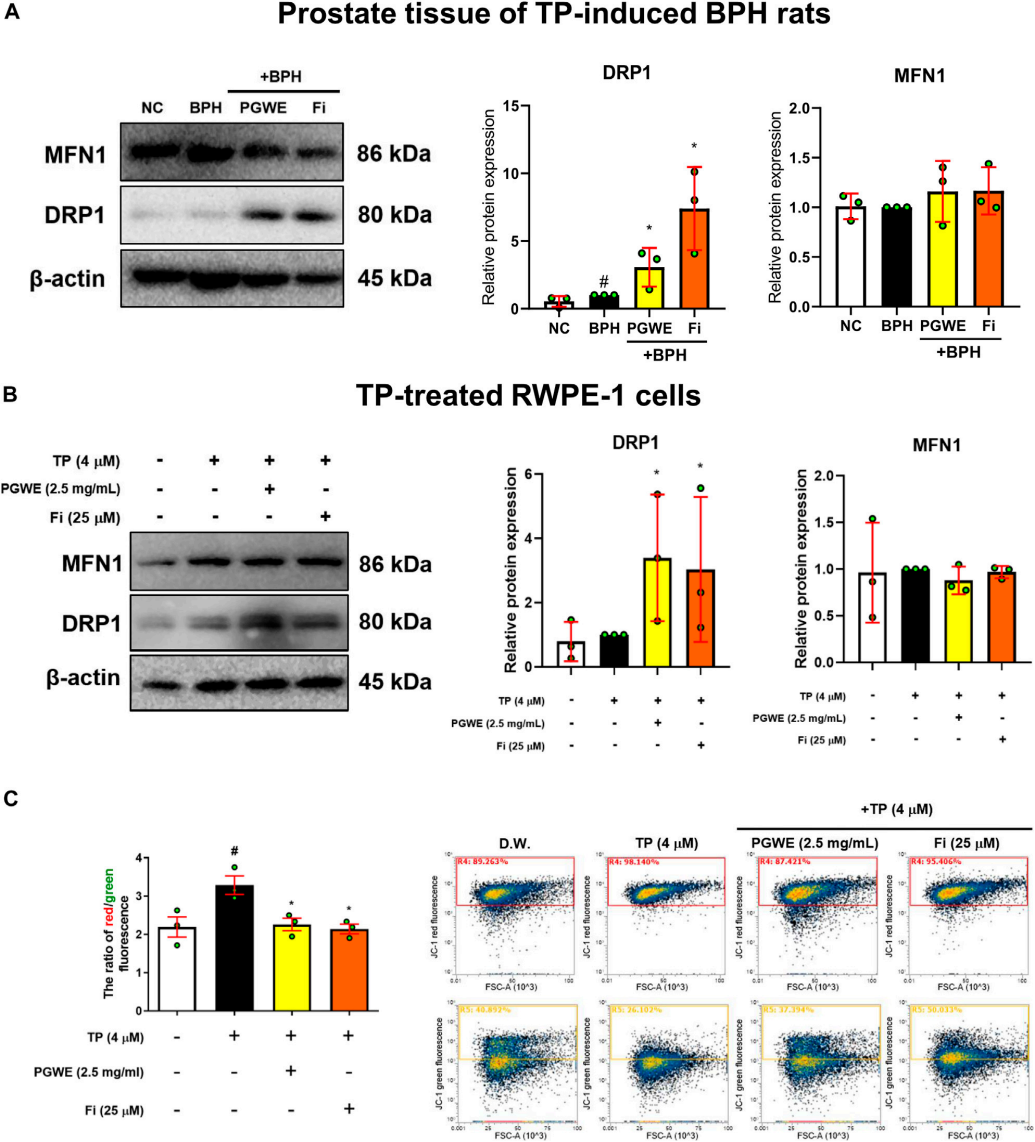
PGWE increases DRP1 level in prostate tissue of BPH rats and TP-treated RWPE-1 cells **(A)** The expressions of MFN1 and DRP1 in prostate tissue of TP-induced BPH rats were analyzed using Western blotting **(B)** The expressions of MFN1 and DRP1 in TP-treated RWPE-1 cells were analyzed using Western blotting and β-actin was used as the loading control **(C)** RWPE-1 cells were stained with JC-1 and analyzed with flow cytometry. All data are expressed as mean ± SEM of data from independent experiments. Statistical differences were evaluated using an unpaired *t*-test and a subsequent post-hoc one-tailed *Mann-Whitney U* test. ^#^ *p* < 0.05 vs NC groups or D.W.-treated RWPE-1 cells; ^∗^ *p* < 0.05 vs BPH group or TP-treated RWPE-1 cells. NC, normal control; BPH, benign prostatic hyperplasia; TP, testosterone propionate; PGWE, *Panax ginseng* C. A. Meyer water extract; Fi, finasteride; D.W., distilled water.

**FIGURE 5:**
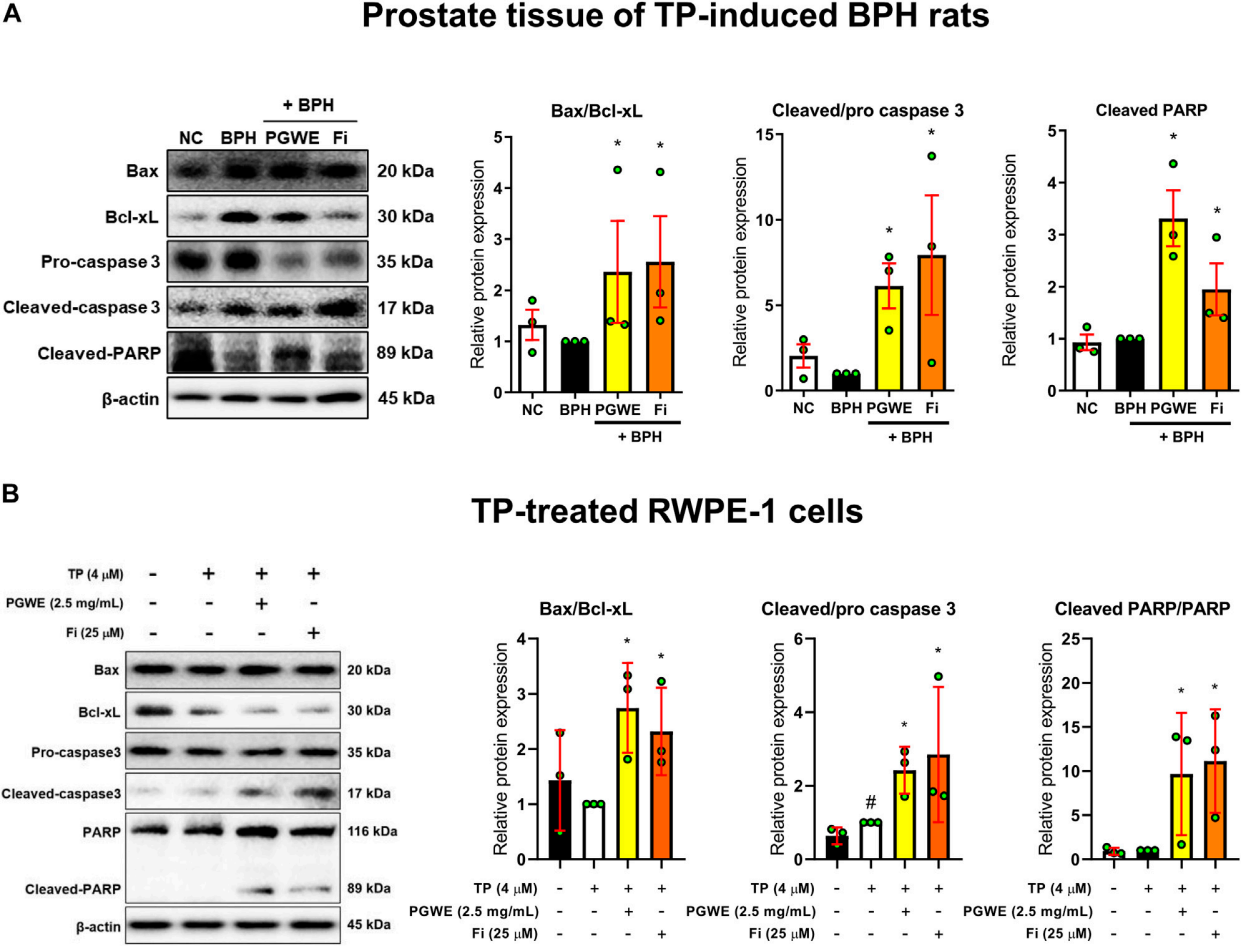
PGWE induces apoptosis in prostate tissue of BPH rats and TP-treated RWPE-1 cells **(A)** The expressions of Bax, Bcl-xL, caspase-3, and PARP in prostate tissue of TP-induced BPH rats were analyzed using Western blotting **(B)** The expressions of Bax, Bcl-xL, caspase-3, and PARP in TP-treated RWPE-1 cells were analyzed using Western blotting and β-actin was used as the loading control. All data are expressed as mean ± SEM of independent experiments. Statistical differences were evaluated using an unpaired *t*-test and a subsequent post-hoc one-tailed *Mann-Whitney U* test. ^#^ *p* < 0.05 vs. NC groups or D.W.-treated RWPE-1 cells; ^∗^ *p* < 0.05 vs. BPH group or TP-treated RWPE-1 cells. NC, normal control; BPH, benign prostatic hyperplasia; TP, testosterone propionate; PGWE, *Panax ginseng* C. A. Meyer water extract; Fi, finasteride; D. W., distilled water.

To investigate whether PGWE regulates mitochondrial dynamics in development of BPH, we identified the key regulators of mitochondrial dynamics (Figures 4A, B). PGWE increased DRP1, a regulator of mitochondrial fission, without affecting MFN1, a mitochondrial fusion regulator, in both prostate tissues of rats and RWPE-1 cells. We also evaluated mitochondrial viability and function using a fluorescent cationic probe JC-1. As shown in Figure 4C, TP increased the ratio of red/green fluorescence and PGWE reduced the ratio (3.18 ± 0.1 and 2.1 ± 0.1, respectively) to a level similar to that of Fi (2.1 ± 0.05) in RWPE-1 cells. From the result that PGWE induces the loss of electrochemical potential, we inferred that PGWE could induce apoptosis. Therefore, we further checked the expressions of Bax and Bcl-xL, the representative factors of apoptosis, to confirm the effects of PGWE on apoptosis. As shown in Figure 5A, PGWE or Fi treatments increased not only the ratio of Bax/Bcl-xl but also the protein levels of caspase three and PARP, other factors included in the apoptosis signaling pathway, in prostate tissues of the BPH-induced rats. Consistently PGWE showed an apoptotic effect in TP-treated RWPE-1 cells (Figure 5B).

### Effect of PGWE against the reduction of sperm count induced by Fi

**FIGURE 6:**
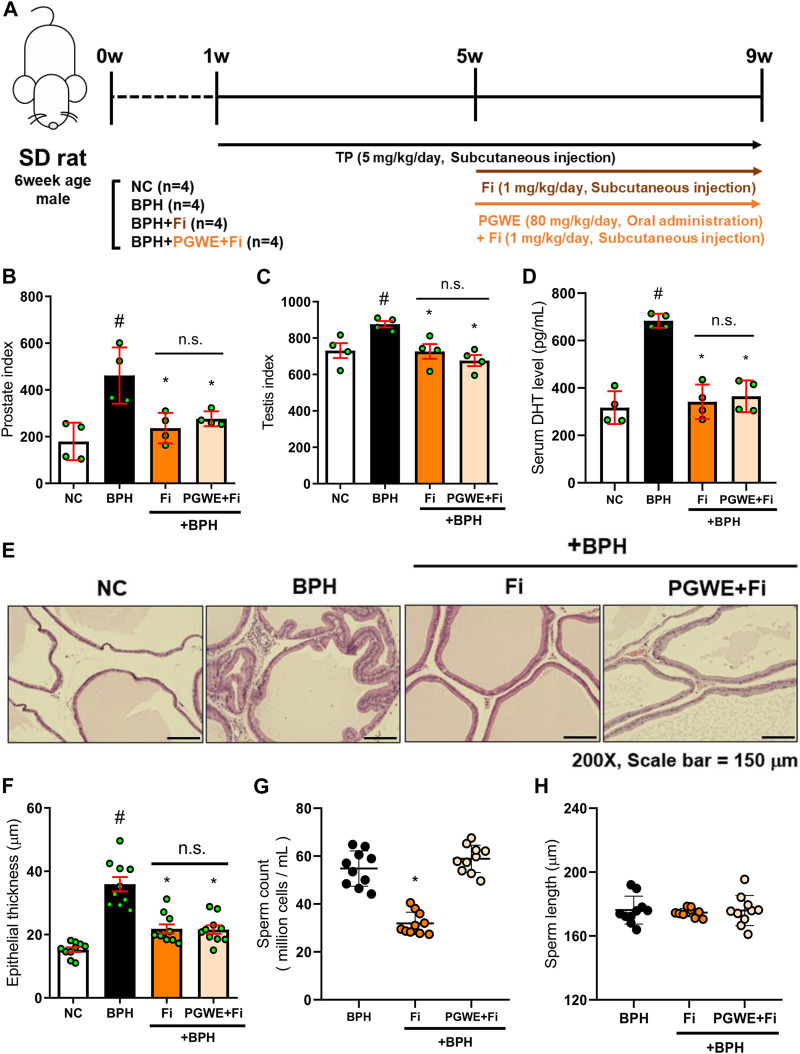
PGWE restores loss of sperm count by Fi **(A)** Experimental scheme of *in vivo* study **(B)** The prostate index was calculated by prostate weight (mg)/body weight (100 g) **(C)** The testis index was calculated by testis weight (mg)/body weight (100 g) **(D)** Serum DHT levels were analyzed using ELISA kits **(E)** H and E staining analysis was performed using prostate tissues of TP-induced BPH rats (magnification × 200, scale bar 150 µm) **(F)** Based on H&E staining, the epithelial thickness was measured using ImageJ software **(G)** Number of sperm extracted from the epididymis of rats and **(H)** length of sperm were calculated using ImageJ software. All data are expressed as mean ± SEM of independent experiments. Statistical differences were calculated by one-way ANOVA with post-hoc Tukey’s test. ^#^ *p* < 0.05 vs. NC group; ^∗^ *p* < 0.05 vs. BPH group. TP, testosterone propionate; NC, normal control; BPH, benign prostatic hyperplasia; PGWE, *Panax ginseng* C. A. Meyer water extract; Fi, finasteride.

To confirm if PGWE could alleviate the side effects of Fi, we administered Fi and PGWE in combination to the BPH-induced rats. The detailed experimental scheme is shown in Figure 6A. As expected, Fi administration reduced prostate and testis index and relieved pathological signs such as serum DHT level and prostate epithelial thickness (Figures 6B–F). However, the sperm count in the epididymis was significantly decreased by Fi (Figure 6G). Interestingly, this reduction in sperm count was improved to the basal level by PGWE. Compared to the Fi group, the PGWE + Fi group did not show the reduction of sperm count induced by Fi. On the other hand, there is no difference in the length of the sperm between the two groups (Figure 6H).

## Discussion

BPH, characterized by abnormal proliferation of the prostate, is a common disease in adult men (Langan, 2019). Although the exact mechanism of BPH has not been identified, several studies have reported that androgens are closely related to the pathogenesis of BPH (Nicholson and Ricke, 2011). Therefore, regulation of the AR signaling pathway is an important target in the treatment of BPH (Roper, 2017). Testosterone in the prostate is converted into DHT by 5α-reductases. Then DHT binds with AR. Upon binding of androgens, AR is translocated into the nucleus to bind androgen response elements, resulting in the activation of genes encoding various growth factors (Grino et al., 1990). Based on these mechanisms, 5α-reductase inhibitors inhibit the conversion of testosterone to DHT through inhibition of the 5α-reductase enzyme (Kaplan et al., 2008). However, it is reported that 5α-reductase inhibitors, including Fi and dutasteride, cause serious side effects such as erectile dysfunction, loss of libido, and impaired ejaculation (Carreño-Orellana et al., 2016). For this reason, natural medicines have been attracting attention as new candidate therapeutic agents to replace existing synthetic drugs. Our study aims to verify that PGWE derived from natural products has therapeutic effects on BPH. PGWE alleviated various pathological signs of BPH by reducing the size and epithelial thickness of the prostate, and by decreasing the serum level of DHT in the TP-treated BPH rat model. In addition, PGWE regulated AR-mediated proliferation by suppressing the protein expression of 5AR2 and AR in prostate tissues of TP-induced BPH rats and TP-treated RWPE-1 cells. These results suggest that PGWE has the potential to be a new alternative treatment for BPH.

Cell apoptosis is an important mechanism for preventing abnormal cell proliferation because it regulates cell growth (Gavrilescu and Denkers, 2003). Apoptosis proceeds through intrinsic and extrinsic pathways (Indran et al., 2011). In particular, the intrinsic apoptotic pathway is activated by various microenvironmental changes such as reduction of growth factors, DNA damage, ER stress, active oxygen overload, and DNA replication stress (Loreto et al., 2014). Drp1-mediated apoptosis also occurs through the intrinsic apoptotic pathway (Suen et al., 2008). As mitochondrial fission increases during apoptosis, mitochondria undergo extensive fragmentation, and some proteins related to mitochondrial dynamics are directly involved in the regulation of apoptosis (Oettinghaus et al., 2016; Milani et al., 2019). Excessive mitochondrial fission also increases the release of cytochrome C by Bax translocation and activation (Estaquier and Arnoult, 2007). Cytochrome C released into the cytoplasm binds to apoptotic peptide activating factor1 (Apaf1) and pro-caspase nine to form a protein complex known as apoptosome, through which caspase nine is activated. Activated caspase nine generates cascades that activate substances involved in cell apoptosis (Ow et al., 2008). In this study, we confirmed that PGWE increased DRP1 both in prostate tissues of TP-induced BPH rats and in TP-treated RWPE-1 cells. In addition, it was confirmed that PGWE increases not only the Bax/Bcl-xL ratio but also protein levels of cleaved caspase-3 and cleaved PARP. As in the tissues, the ratio of Bax/Bcl-xL and protein expression of cleaved caspase-3 and cleaved PARP were increased by PGWE treatments in TP-treated RWPE-1 cells. These data mean that PGWE alleviates BPH by inducing drp1-mediated intrinsic apoptosis.

Finasteride is a 5α-reductase inhibitor that has been approved by the FDA for the treatment of benign prostatic hyperplasia (Cather et al., 1999). It has previously been considered a safe drug with few side effects, but recently drug-related adverse reactions (such as decreased libido, erection disorders, and sperm loss) that persist long enough to give rise to the term post-finasteride syndrome have been reported (Pereira and Coelho, 2020). Therefore, although finasteride is the most effective treatment for benign prostatic hyperplasia, many patients are reluctant to take it due to side effects. In our experiments, a decrease in sperm count was confirmed in the Fi group. However, PGWE did not affect sperm count while improving BPH as much as Fi does. Surprisingly, PGWE protects against Fi-induced sperm loss when co-administered with Fi. Consequently, PGWE not only relieves the symptoms of BPH but also improves the side effects of Fi. Further study is needed on how PGWE manages the side effects of Fi, but these results show that PGWE alone is sufficient as a new substitute for BPH treatment.

## Data Availability

The original contributions presented in the study are included in the article/Supplementary Materials, further inquiries can be directed to the corresponding author.

## Abbreviations

AR; androgen receptor, BAX; Bcl-2-associated X protein, Bcl-xL; B-cell lymphoma extra-large, BPH; benign prostatic hyperplasia, DHT; dihydrotestosterone, DRP1; dynamin-related protein one, DW; distilled water, Fi; finasteride, MFN1; mitofusin-1, NC; normal control group, PGWE; water extract of Panax ginseng CA. Meyer. PSA; prostate-specific antigen, TP; testosterone propionate, 5AR2; five alpha reductases two.
