# Supplementary figures and images for: Panax ginseng C.A. meyer alleviates benign prostatic hyperplasia while preventing finasteride-induced side effects
Source: Front Pharmacol. 2023 Jan 12;14:1039622. doi: 10.3389/fphar.2023.1039622 (PMC9877295; doi:10.3389/fphar.2023.1039622)

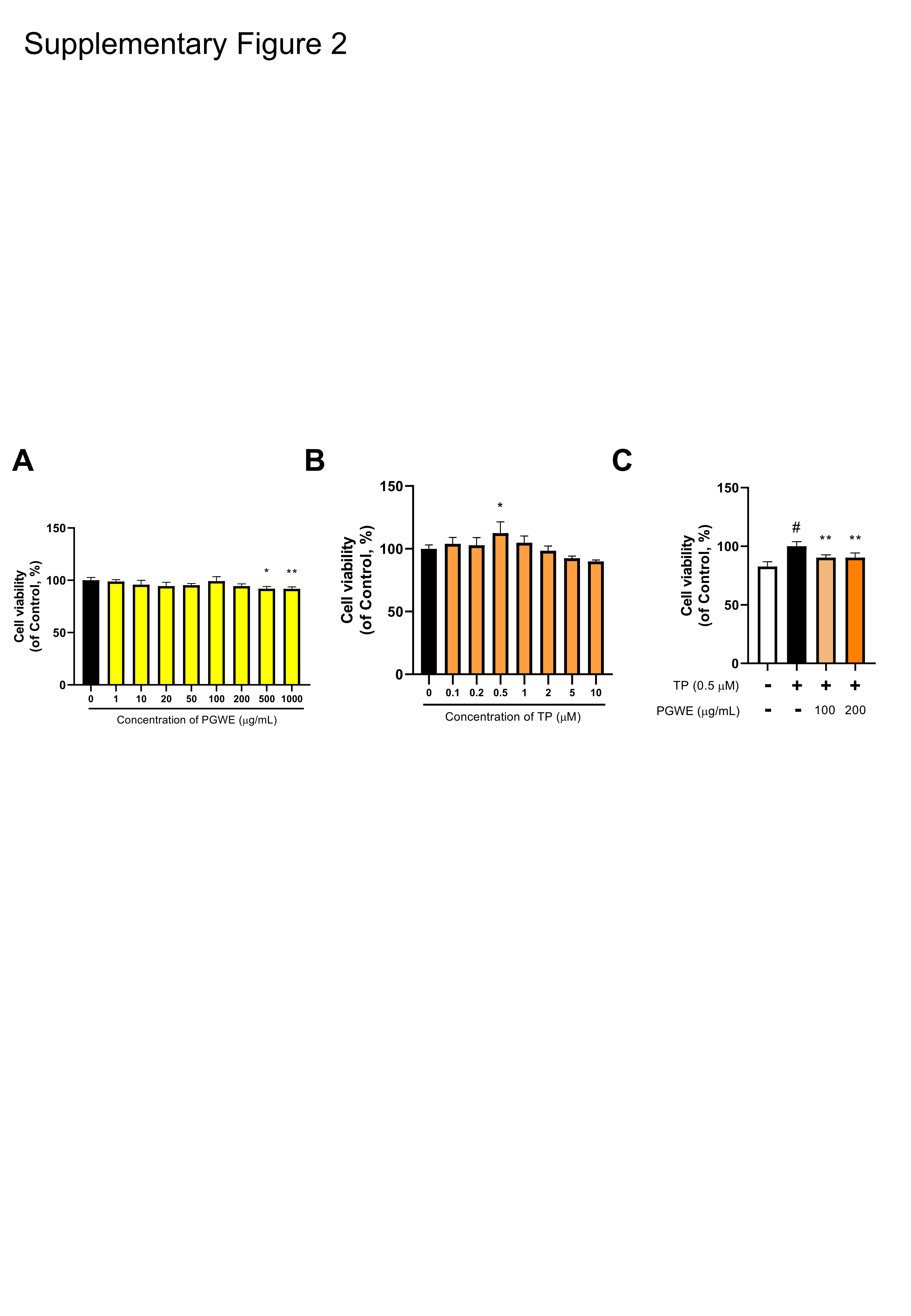

Supplement: Supplementary file 1 [file Image2.TIF]

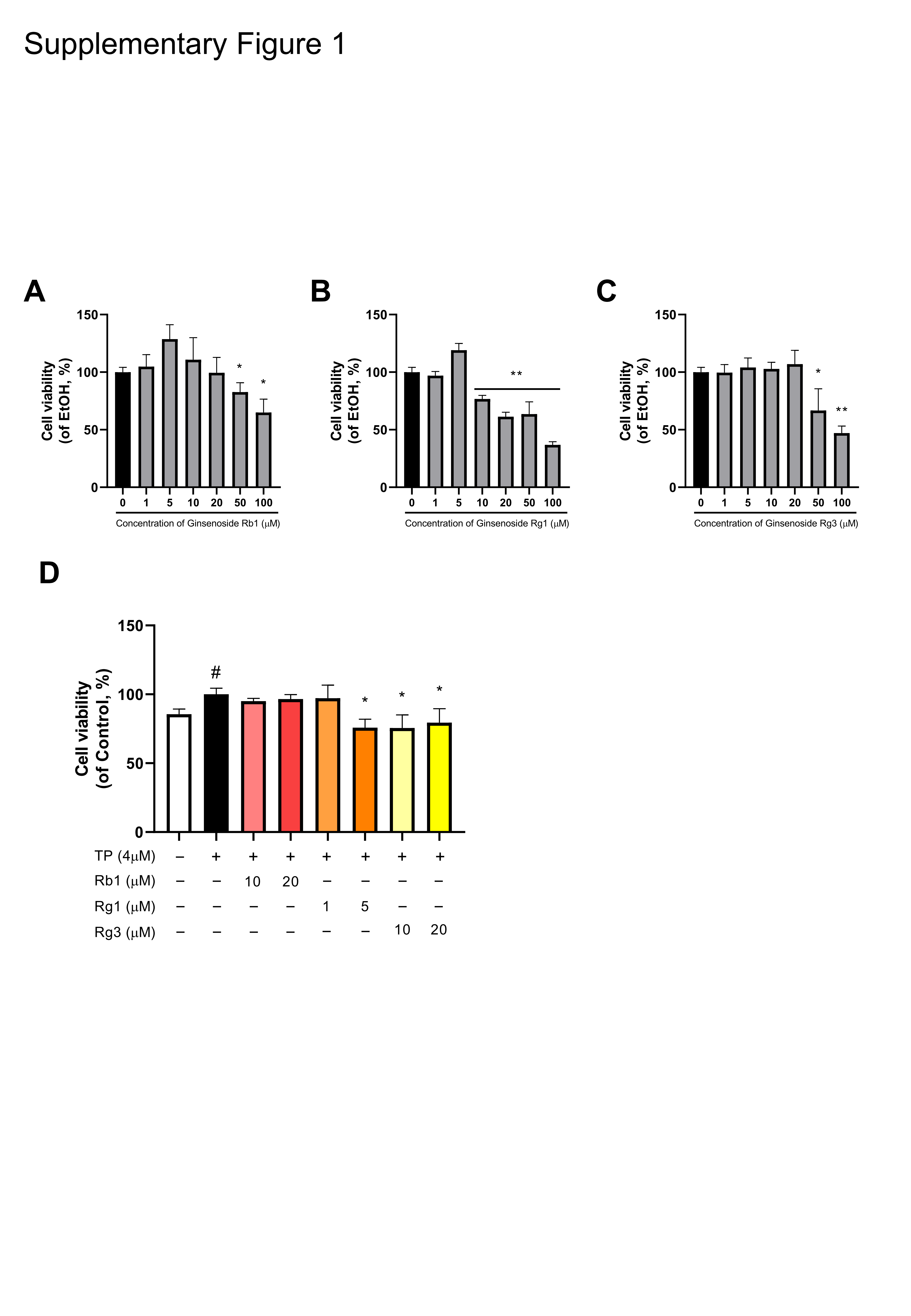

Supplement: Supplementary file 2 [file Image1.TIF]
