# Supplementary material for: Panax ginseng C.A. meyer alleviates benign prostatic hyperplasia while preventing finasteride-induced side effects
Source: Front Pharmacol. 2023 Jan 12;14:1039622. doi: 10.3389/fphar.2023.1039622 (PMC9877295; doi:10.3389/fphar.2023.1039622)
Supplement: Supplementary file 3 [file DataSheet1.docx]

**Data in Brief (supplementary figures)**

*Panax ginseng* C.A. Meyer alleviates benign prostatic hyperplasia while preventing finasteride-induced side effects

Park et al.

Supplementary Figure 1. Ginsenoside Rg1 and Rg3 suppress cell proliferation in TP-treated RWPE-1 cells.

Supplementary Figure 2. PGWE suppresses cell proliferation in TP-treated WPMY-1 cells.

**Supplementary Figure 1. Ginsenoside Rg1 and Rg3 suppress cell proliferation in TP-treated RWPE-1 cells.**

(A), (B), (C) Cell viability of Ginsenoside Rb1, Rg1 and Rg3 were measured by WST-1 assay. (B) Cell viability of Ginsenoside Rb1, Rg1 and Rg3 on TP (4 μM)-treated RWPE-1 cells was measured by WST-1 assay. All data are expressed as the mean ± S.E.M. of three or more separate measurements. ^#^*p* < 0.05 vs. D.W.-treated RWPE-1 cells; ^*^*p* < 0.05 and ^**^*p* < 0.01 vs. TP-treated RWPE-1 cells. TP, testosterone propionate; PGWE, *Panax ginseng* C.A. Meyer water extract; D.W., distilled water.

**Supplementary Figure 2. PGWE suppresses cell proliferation in TP-treated WPMY-1 cells.**

(A) Cell viability of PGWE was measured by WST-1 assay. (B) Cell viability of TP was measured by WST-1 assay. (C) Cell viability of PGWE on TP (0.5 μM)-treated WPMY-1 cells was measured by WST-1 assay. All data are expressed as the mean ± S.E.M. of three or more separate measurements. ^#^*p* < 0.05 vs. D.W.-treated WPMY-1 cells; ^∗^*p* < 0.05 and ^**^*p* < 0.01 vs. TP-treated WPMY-1 cells. TP, testosterone propionate; PGWE, *Panax ginseng* C.A. Meyer water extract; D.W., distilled water.
